# Cutting out the φC31 prophage

**DOI:** 10.1111/j.1365-2958.2011.07699.x

**Published:** 2011-06

**Authors:** W Marshall Stark

**Affiliations:** Institute of Molecular, Cell and Systems Biology, University of GlasgowGlasgow G12 8QQ, UK

## Abstract

To establish a lysogenic lifestyle, the temperate bacteriophage φC31 integrates its genome into the chromosome of its *Streptomyces* host, by site-specific recombination between *attP* (the attachment site in the phage DNA) and *attB* (the chromosomal attachment site). This reaction is promoted by a phage-encoded serine recombinase Int. To return to the lytic lifestyle, the prophage excises its DNA by a similar Int-mediated reaction between the recombinant sites flanking the prophage, *attL* and *attR*. φC31 Int has been developed into a popular experimental tool for integration of transgenic DNA into the genomes of eukaryotic organisms. However, until now it has not been possible to use Int to promote the reverse reaction, excision. In many other phages, the presence of a recombination directionality factor (RDF) protein biases the phage-encoded integrase towards prophage excision, whereas absence of the RDF favours integration; but the φC31 RDF had proved elusive. In this issue of *Molecular Microbiology*, Khaleel *et al*. (2011) report the identification and purification of the φC31 RDF, and show that it both promotes excision and inhibits integration by direct protein-protein interactions with Int itself.

Virulent bacteriophages destroy their host cell as they replicate and release new phage particles into the surrounding medium, but many ‘temperate’ phages can also adopt a lysogenic lifestyle where they hide in the host cell and get passed on from generation to generation. The iconoclastic discovery that phage λ integrates its DNA into the host genome to establish lysogeny was followed by a surge of groundbreaking research on the integration mechanism. It was revealed that the phage uses a site-specific recombinase enzyme (Int) to paste its circular DNA genome into the chromosome of the bacterial cell, by breaking and rejoining the strands at two sites called *attP* (on the phage) and *attB* (on the chromosome). The so-called prophage can remain quietly embedded in the host cell's DNA for thousands of generations, but eventually, to re-enter the lytic lifestyle and produce more infectious particles, the phage genome must be cut out and recircularized. Both excision and integration are promoted by the same enzyme, Int, but how does it know which way to go? Phage λ controls directionality with a protein called Xis. In the absence of Xis, Int promotes the *attB* × *attP* integration reaction, but has no activity on the recombinant *attL* and *attR* sites that flank the prophage DNA. However, in the presence of Xis the reverse is true; Int promotes *attL* × *attR*, but not *attP* × *attB* recombination. This switch is brought about by binding of Xis to the phage-encoded recombination site DNA; binding to *attP* inhibits integration, whereas binding to *attR* strongly stimulates excision. It is thought that Xis modulates Int activity by distorting the path of the DNA within the elaborate protein-DNA complexes formed when two *att* sites come together (Azaro and Landy, 2002).

λ Int was the first site-specific recombinase to be characterized. We now know of many hundreds of related enzymes, which form a group known as the tyrosine recombinases – so-called because a conserved tyrosine residue is the active site nucleophile that breaks the phosphodiester of the DNA backbone, becoming covalently attached to the DNA in the process. Many other phages can adopt a lysogenic lifestyle like λ, and for a long time all known phage integrases were tyrosine recombinases. However, in the 1990s it was discovered that some temperate phages, including φC31, use integrases belonging to a very different family of enzymes, the serine recombinases (Smith and Thorpe, 2002; Smith *et al*., 2010). Serine recombinases were already known as a close-knit family of small proteins (∼ 200 amino acids) that promote programmed genetic rearrangements such as resolution of transposition intermediates, plasmid monomerization, and inversion of the orientation of genomic DNA segments. They use a serine residue rather than a tyrosine to break the DNA strands, and are structurally and mechanistically unrelated to the tyrosine recombinases (Grindley *et al*., 2006). The newly identified phage integrases had an N-terminal domain that could be aligned with the known serine recombinases, but they were much larger proteins, typically 500–800 amino acids. The functions of the large C-terminal extensions were unknown at first. As φC31 Int and related systems were characterized, initially *in vivo* and then *in vitro* (Thorpe and Smith, 1998; Thorpe *et al*., 2000), a curious difference from the λ-like systems became apparent; whereas λ Int promotes recombination at a large, complex phage *attP* site (∼ 240 bp), both the *attP* and *attB* sites for serine integrases are short (30–40 bp), with imperfect dyad symmetry suggesting that each site might just bind a dimer of Int. Furthermore, the *in vitro* studies showed that φC31 Int is the only protein that is required for *attP*×*attB* recombination, whereas λ Int requires an additional protein IHF. It was not at all clear how serine integrases could control directionality with such simple recombination sites (Smith and Thorpe, 2002). And yet, φC31 Int did not promote recombination between *attL* and *attR in vitro*. The experiments implied that an excision-specific factor was missing.

The simplicity of the recombination sites used by φC31 Int and its capacity for one-way integration immediately attracted the attention of those working in the nascent field of genome engineering. As a result, φC31 Int has become a recombinase of choice for efficient integration of transgenic DNA into specific genomic loci of model organisms (Calos, 2006). The opportunities for application of this system might be expanded considerably if it could promote unidirectional excision as well as integration.

The first ‘RDF’ (recombination directionality factor, as proteins with Xis-like function have become known) of a serine integrase was identified in 1999 in phage TP901-1 (Breuner *et al*., 1999). Two other serine integrase RDFs followed, and it became apparent that phages can use a variety of unrelated proteins for this purpose. The discovery of proteins related to these RDFs in phages that do not have serine integrases has led to the suggestion that at least some RDFs have alternative functions, and have been co-opted by the integrase system to regulate directionality. Prior to the work of Khaleel *et al*. (2011), the best understood serine integrase RDF was that of the *Mycobacterium* phage Bxb1 (gp47). gp47 was purified and shown to interact directly with Bxb1 Int *in vitro*, forming a stable complex when Int is itself bound to an *att* site (Ghosh *et al*., 2006; 2008), unlike Xis from phage λ, which can bind to *att* sites independently of Int. Meanwhile however, the φC31 RDF was proving hard to catch; no proteins in the φC31 genome resembled any of the previously identified RDFs.

Khaleel *et al*. guessed that the φC31 RDF might interact directly with Int, like the BxbI RDF. They therefore set out to test a set of 28 φC31-encoded proteins to see if any of them bound to Int in an *E. coli*-based ‘two-hybrid’ assay. Only two proteins (other than Int itself) were recovered from this screen, and of these one, a 27.5 kDa protein called gp3, turned out to be the elusive RDF. The two-hybrid assays also showed that gp3 subunits interact, suggesting that it might be functionally multimeric. Recombination assays showed that gp3 dramatically stimulated Int-mediated *attL* × *attR* recombination and inhibited *attP* × *attB* recombination. Intriguingly, gp3 also stimulated recombination between two *attL* sites or two *attR* sites but not other combinations of *att* sites, lending support to the proposal (Ghosh *et al*., 2008) of a ‘rule’ that in a productive pairing of sites, each Int subunit bound to an *attP*-derived half-site must pair with an Int bound to an *attB*-derived half-site, and *vice versa*.

To gain more detailed insight into the effects of gp3, the authors purified the protein. Using a ‘pulldown’ assay with gp3 immobilized on beads, they showed that it bound specifically to Int, even in the absence of any *att* site DNA. They then studied the complexes formed by gp3 together with Int and the *att* sites using electrophoretic mobility shift assays (EMSA). These experiments revealed a number of important features. First, there was no interaction of gp3 with the *att* site DNA in the absence of Int, suggesting that unlike λ Xis, gp3 may act entirely by protein-protein interactions. Int on its own forms monomer and dimer complexes with all the *att* sites, and can also be observed to make a synapse between *attP* and *attB*, but not between *attL* and *attR*. When gp3 and Int are both present, new, lower-mobility complexes of single *att* sites are observed, presumed to contain subunits of both proteins. But most importantly, a synapse between *attL* and *attR* can now be observed, while formation of the *attP* × *attB* synapse is inhibited. Thus, gp3 acts at the synapsis stage of recombination, to favour the excision synapse and disfavour the integration synapse. In accord with the results of the recombination assays in *E. coli*, synapsis of two *attL* sites or two *attR* sites could also be detected, but no synapses were seen between other combinations of sites.

Is the RDF (gp3) present in the synaptic complexes, or does it act by remodelling the Int-DNA complexes and then dissociating? Previously, the Smith group had isolated an Int mutant that can synapse and recombine either *attP* × *attB* or *attL* × *attR* without gp3. Using this mutant, it was shown that the electrophoretic mobilities of both attP-attB and attL-attR synaptic complexes are shifted in the presence of gp3, suggesting that gp3 is in the complexes. Furthermore, a kinetic analysis of the excision reaction suggested a stoichiometric interaction of gp3 with Int.

Figure 1 summarizes our understanding of the mechanisms of φC31 Int-mediated integration and excision, following the work of Khaleel *et al*. Their results now provide us with all the pieces of the jigsaw needed to study the complete system in molecular detail. We still have a lot to learn about this fascinating family of enzymes, including the stoichiometry of the synaptic complexes and the nature of the proposed RDF-induced structural changes. And of course, we eagerly await crystallography!

**Fig. 1:**
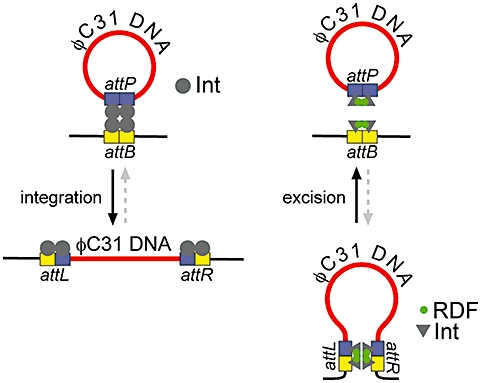
Mechanism of φC31 integration and excision. Int subunits are represented by grey circles (for integration) or triangles (for excision), to indicate the proposed protein conformational changes. Note that the actual stoichiometry of the Int and RDF subunits remains unknown. Black and red lines represent the host and phage genomic DNA.
